# P324: Management of risk in a country in crisis: hepatonephritis cases related to artemisinin- based combinations therapy

**DOI:** 10.1186/2047-2994-2-S1-P324

**Published:** 2013-06-20

**Authors:** T Daubrey Potey, M Kamagaté, H Dié-Kacou

**Affiliations:** 1 UFR des Sciences pharmaceutiques, Université Felix Houphouët Boigny, Abidjan, Côte d'Ivoire

## Objectives

To analyze risk management when occurred hepatonephritis cases related to Artemisinin-based Combinations Therapy.

## Methods

A technical committee of management of hepatonephritis cases has been set up by the health ministry. The terms of references were: 1) state of knowledges on the subject from the pharmacovigilance database of Côte-d’Ivoire; 2) Carrying out of an investigation in public and private hospitals; 3) start -up a plan of communication for the health professionals and population.

## Results

**Results**: States of knowledges: for 10 years, serious Adverse Drug reactions have been notified (57%), like black fever, hepatonephritis and serious hepatitis cases related to any antimalarial monotherapies : amodiaquin, artemisinin derivatives and quinine. Retrospective study: we collected 100 files. The principal damage was hepatic (63%), hepatorenal (26 %) and hematologic (19%). Antimalarial drugs implicated were: quinine (30,4%), artemether-lumefantine (21,4%) and artesunate-sulfamethopyrazine (13,6%)..Prospective study: We have analyzed 25 cases over 3 months of investigation.

## Conclusion

**Conclusion**: Start-up of a plan of communication: we drafted and sent an alarm note and recommendations of good use of antimarial combinotherapies to prescribers and dispensers.

## Disclosure of interest

None declared

